# Clinical Dilemmas in Immune Thrombocytopenic Purpura With Diffuse Alveolar Hemorrhage: Diagnosis, Treatment, and Outcomes

**DOI:** 10.7759/cureus.47300

**Published:** 2023-10-18

**Authors:** Saleh A Ba-Shammakh, Eman A Al-Zughali, Zeina H Kalaji, Abdulrahman M Al-Bourah, Nashaat A Al-Shami

**Affiliations:** 1 Department of General Surgery, The Islamic Hospital, Amman, JOR; 2 Department of General Surgery, Princess Rahma Teaching Hospital, Irbid, JOR; 3 Department of Internal Medicine, The Islamic Hospital, Amman, JOR; 4 Faculty of Medicine, The University of Jordan, Amman, JOR

**Keywords:** complication, splenectomy, diffuse alveolar hemorrhage, dah, itp, idiopathic thrombocytopenic purpura

## Abstract

This report elucidates a unique case of a 39-year-old female with immune thrombocytopenic purpura (ITP) who developed a rare and severe complication: diffuse alveolar hemorrhage (DAH). Despite initial treatments for ITP, the patient experienced fluctuating platelet (PLT) counts and shortness of breath, which were later identified as symptoms of DAH. An urgent splenectomy improved the patient's platelet counts and overall condition. This case underscores the imperative to recognize DAH as a possible ITP complication, requiring clinicians' vigilance for prompt diagnosis and intervention. The intricate nature of ITP in adults necessitates individualized, patient-centered treatment approaches to enhance outcomes. This report provides invaluable insights into the clinical understanding and management of ITP and its complications through detailed analysis and documentation of the patient's treatment journey.

## Introduction

Immune thrombocytopenic purpura (ITP), previously known as idiopathic thrombocytopenic purpura, is commonly diagnosed when platelet (PLT) counts fall below 100,000/uL. However, clinical symptoms usually manifest at even lower platelet counts. This disorder is characterized by the immune-mediated destruction of platelets, primarily through the reticuloendothelial system, and can manifest in various forms depending on its duration and the patient's age [1,2].

In the United States, the annual incidence rate of ITP is 3.3 per 100,000, while in Europe, it varies between 1 and 4 per 100,000 depending on the country. Preliminary studies in the Middle East and North Africa (MENA) region aim to characterize the epidemiology of ITP, with findings showing a female predominance and a mean age of 28.29 (±17.34) years in the Africa, Middle East, and Asia (AMEA) regions [3]. While childhood ITP is generally a benign, self-limiting disease affecting both genders equally, adult ITP has a predominant incidence in females (80%) and is often chronic, with an increased frequency of complications [1,4].

Over the past decade, our understanding of ITP's pathophysiology has evolved. We now know that it involves not only antibody-mediated platelet destruction but also impaired platelet production due to megakaryocyte destruction, as well as processes not evidently antibody-mediated [2]. A subset of patients shows no apparent antibody-mediated process, suggesting a potential failure of tolerance to self [2]. The condition can manifest as acute or chronic and can be primary or secondary to other causes, including human immunodeficiency virus (HIV), cancer, autoimmune conditions, hepatitis, or certain medications.

Stratified classifications have been developed to tailor treatment regimens based on age and presentation, helping to avoid unnecessary interventions given the self-resolving nature of ITP in children and the more complex manifestations in adults [5].

ITP's clinical presentations are diverse, ranging from asymptomatic findings to severe outcomes such as genitourinary, gastrointestinal, and intracranial bleeding. In females, heavy menstrual bleeding can also be a symptom [6]. Fatigue is a recognized symptom even in cases previously deemed asymptomatic. It is also noteworthy that ITP patients are at risk for thrombophilia, a condition that requires consideration [6].

Diffuse alveolar hemorrhage (DAH), identified through widespread radiographic alveolar infiltrates, hemoptysis, anemia, and respiratory failure, is rarely associated with ITP [7]. Only two cases of DAH resulting from ITP have been documented in the English literature, with an additional case reported in Baltimore. This scarcity of reports highlights the critical need for medical professionals to acknowledge DAH as a possible complication of ITP [8]. Identifying DAH requires careful examination, looking for indicators such as increasing amounts of bloody fluid obtained from consecutive bronchoalveolar lavages, macrophages filled with hemosiderin identified through cytological studies, and a thorough collection of patient history, physical examination, and laboratory tests [9]. The case discussed in this report involves a 39-year-old female with persistent ITP that became complicated due to the onset of DAH, underlining the importance of clinician vigilance and awareness of the potential linkage between ITP and DAH, given the rarity and seriousness of this complication.

## Case presentation

A 39-year-old female was diagnosed with idiopathic thrombocytopenia (ITP) in May 2023 in Yemen, presenting with symptoms of epistaxis, heavy menstrual bleeding, and skin bleeding spots. The diagnosis was incidentally discovered through a complete blood count (CBC) performed before labor, indicating a platelet count of 48,000 × 10^9^/L. Despite treatment with steroids, intravenous immunoglobulin (IVIG), azathioprine, eltrombopag, and four doses of infliximab (with the last administered on August 21, 2023), her platelet count would transiently increase before plummeting again. A bone marrow biopsy in Yemen further confirmed the ITP diagnosis.

During a recent admission to our department, the patient reported experiencing shortness of breath for three days with a gradual onset, progressively worsening and exacerbated by exertion. This symptom was partially relieved by rest, increased at night, and was described as feeling like choking. The shortness of breath was unrelated to body position and did not awaken her from sleep. She reported no chest pain, palpitations, cough, sore throat, or nasal congestion. There was no audible wheezing or lower limb swelling.

Upon physical examination, a purpuric, non-blanchable rash was observed, with the following vital signs noted: blood pressure (BP) of 126/83 mmHg, heart rate (HR) of 100 beats/minute, respiratory rate (RR) of 21 breaths/minute, and O2 saturation of 89% on room air. Laboratory results highlighted a critical platelet count of <5,000 × 10^9^/L, hemoglobin (HB) of 12.5 g/dL, white blood cell (WBC) count of 7.1 × 10^9^/L, C-reactive protein (CRP) of 23 mg/L, D-dimer of 0.4 mg/L, and random blood sugar (RBS) of 577 mg/dL (Table 1).

**Table 1:** Laboratory results The table outlines the laboratory results of the patient. Key findings (bold values) include low platelet count, high CRP, LDH, and RBS, indicative of ITP and newly diagnosed type 2 DM. The remaining tests are normal or negative. HB: hemoglobin, WBC: white blood cells, CRP: C-reactive protein, RBS: random blood sugar, TFT: thyroid function test, ANA: antinuclear antibody, RF: rheumatoid factor. HBsAg: hepatitis B surface antigen, HCV: hepatitis C virus, HIV: human immunodeficiency virus, LFT: liver function test, KFT: kidney function test, LDH: lactate dehydrogenase, ITP: immune thrombocytopenic purpura, DM: diabetes mellitus

Laboratory test	Result	Reference range
Platelet count	<5,000 × 10^9^/L	150,000-450,000 × 10^9^/L
HB	12.5 g/dL	12.1-15.1 g/dL (female)
WBC	7.1 × 10^9^/L	4-11 × 10^9^/L
CRP	23 mg/L	<10 mg/L
D-dimer	0.4 mg/L	<0.5 mg/L
RBS	577 mg/dL	70-140 mg/dL
TFT	Normal	-
ANA	Negative	Negative
RF	Negative	Negative
Hepatitis B (HBsAg)	Negative	Negative
Hepatitis C (anti-HCV)	Negative	Negative
HIV	Negative	Negative
LFT	Normal	-
KFT	Normal	-
Acetone	Negative	Negative
LDH	363 U/L	140-280 U/L
Vitamin B12	Normal	200-900 pg/mL

**Figure 1:**
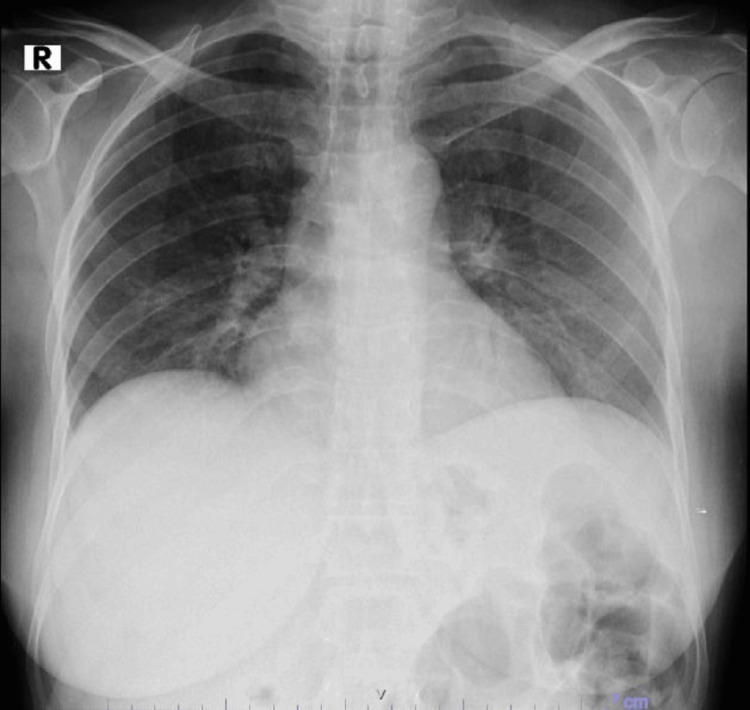
Admission chest X-ray (normal)

A normal chest X-ray was noted (Figure 1). Her medications then included prednisolone, tranexamic acid, norethisterone acetate, and omeprazole. Due to her respiratory complaints, she was started on nasal cannula oxygen therapy at 3 L/minute and treated with ceftriaxone and methylprednisolone while continuing norethisterone acetate and omeprazole. With newly diagnosed diabetes mellitus (DM), an insulin aspart infusion was initiated based on GlucoCheck readings, and an endocrinologist was consulted.

**Figure 2:**
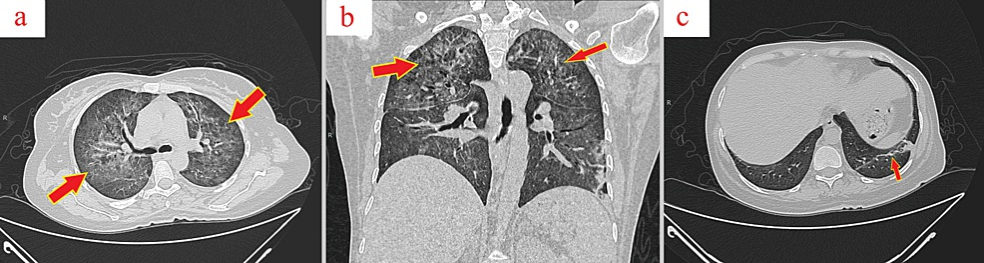
Chest CT scan Chest CT scan reveals confluent ground-glass opacities within both lungs, predominantly in the upper lobes (Figure 2a, 2b) (arrows). A linear atelectatic band is evident in the left lower lobe (Figure 2c) (arrow). These findings are consistent with diffuse alveolar hemorrhage. CT: computed tomography

On the second day of her hospital stay, a high-resolution chest computed tomography (CT) revealed diffuse alveolar hemorrhage (Figure 2), necessitating treatment with pulsed steroids (methylprednisolone 500 mg IV), IVIG (30 g IV), a transfusion of 12 units of platelets, and IV tranexamic acid. Laboratory results showed further deterioration of her condition with PLT of 4,000 × 10^9^/L, HB of 10.7 g/dL, triglycerides (TG) of 284 mg/dL, high-density lipoprotein (HDL) of 17 mg/dL, low-density lipoprotein (LDL) of 120 mg/dL, and hemoglobin A1C (HbA1C) of 10%.

On day 3, the patient received an additional eight units of platelets with other management unchanged. The patient was clinically stable, requiring O2 therapy at 3 L/minute via nasal cannula. Laboratory results were as follows: PLT of 6,000 × 10^9^/L, HB of 9.9 g/dL, WBC of 6.6 × 10^9^/L, and CRP of 20 mg/L, with creatinine (CR), sodium (Na), and potassium (K) all within normal ranges.

On day 4, the patient exhibited signs of oxygen desaturation, necessitating increased oxygen support via a non-rebreather mask at 15 L/minute. Her laboratory results indicated PLT of 39,000 × 10^9^/L, HB of 9.1 g/dL, and WBC of 4.4 × 10^9^/L, with both creatinine (CR) and potassium (K) within normal parameters.

**Figure 3:**
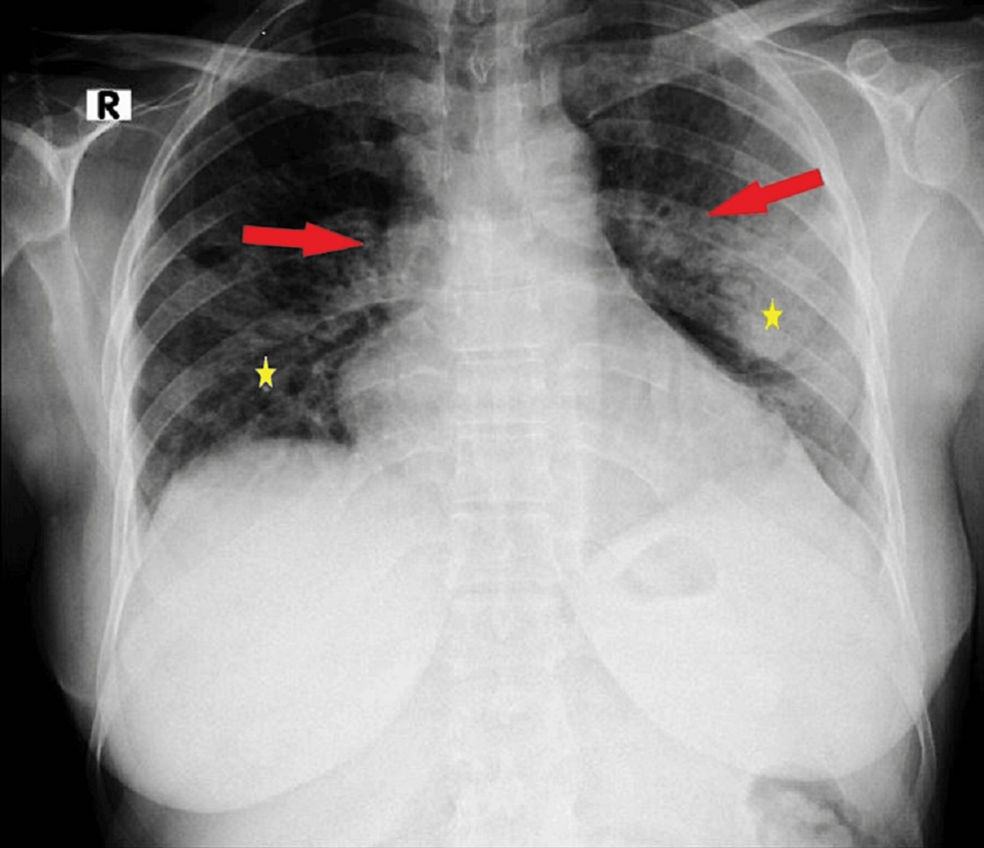
Chest X-ray X-ray demonstrates bilateral perihilar infiltration (stars) and a distinctive "flask-shaped" cardiac silhouette (arrows).

By day 5, she continued to need a non-rebreather face mask. A chest X-ray showed bilateral perihilar infiltration with a "flask-shaped" heart (Figure 3). An echocardiogram (ECHO) revealed an ejection fraction (EF) of 65%, mild left ventricular diastolic dysfunction (LVDD), pulmonary artery systolic pressure (PASP) of 40%-45% (normal: 25%-35%), inferior vena cava (IVC) measuring 1.7 cm (normal: 1.7-2.1 cm), and a mild pericardial effusion with a maximum diameter of 0.3 cm. There was no right atrial (RA) or right ventricular (RV) collapse. IV fluid therapy was halted in response, and furosemide 40 mg IV was administered. IVIG treatment was also stopped. Laboratory results showed PLT of 138,000 × 10^9^/L, HB of 8.5 g/dL, and WBC of 4.3 × 10^9^/L, with creatinine (CR) and potassium (K) remaining normal.

On day 6, the patient still required a non-rebreather mask. Pulsed steroid therapy was discontinued, and she was started on methylprednisolone 40 mg IV every 12 hours. Additionally, she was transitioned to basal-bolus insulin with insulin glargine 25 units subcutaneously every 24 hours and insulin aspart at 20/15/20 units subcutaneously. Laboratory results showed PLT of 290,000 × 10^9^/L, HB of 9.1 g/dL, and WBC of 3.1 × 10^9^/L, with potassium (K) remaining normal.

On day 7, her oxygen needs decreased, and she was maintained on a face mask with oxygen at 5 L/minute. Tranexamic acid was discontinued, and her insulin regimen was modified to insulin glargine 30 units subcutaneously every 24 hours and insulin aspart at 30/30/30 units subcutaneously. Laboratory results displayed PLT of 393,000 × 10^9^/L, HB of 9.4 g/dL, and WBC of 3.9 × 10^9^/L, with potassium (K) remaining normal.

By day 8, the day of her discharge, she appeared well and was set to be released with home oxygen therapy and scheduled for subsequent clinic follow-ups. Laboratory results at discharge showed PLT of 440,000 × 10^9^/L, HB of 9.8 g/dL, and WBC of 3.2 × 10^9^/L. Discharge medications prescribed were esomeprazole 40 mg once daily, norethisterone acetate 10 mg once daily, nystatin three times daily, insulin aspart at 30/30/30 units subcutaneously, insulin glargine 35 units subcutaneously once daily, dapagliflozin/metformin 5/1000 mg orally every 12 hours, and glyburide 5 mg orally once daily. For a comprehensive understanding, Table 2 shows the patient's platelet count fluctuations and key interventions/events during the hospital stay.

**Table 2:** Timeline of platelet counts and interventions Table 2 lists daily platelet counts, transitions, and interventions. *Daily count changes **Decrease from the previous reading CBC: complete blood count, IVIG: intravenous immunoglobulin, IV: intravenous

Day	Platelet count (×10^9^/L)	Platelet transition (×10^9^/L)*	Intervention/event
Pre-diagnosis	48,000	-	Initial CBC before labor
(Admission)	<5,000	-	Critical count during recent admission
Day 2	4,000	-1,000**	Treatment with pulsed steroids, IVIG, transfused 12 units of platelets, IV tranexamic acid
Day 3	6,000	+2,000	Transfused an additional eight units of platelets
Day 4	39,000	+33,000	Increased oxygen support via a non-rebreather mask
Day 5	138,000	+99,000	IVIG treatment stopped, furosemide administered
Day 6	290,000	+152,000	Pulsed steroid therapy stopped, insulin adjusted
Day 7	393,000	+103,000	Oxygen needs decreased, insulin adjusted
Day 8 (discharge)	440,000	+47,000	Patient prepared for discharge with home oxygen therapy
Day 12 (readmission)	14,000	-426,000**	Patient readmitted for urgent splenectomy
Postoperative day 3	168,000	+154,000	Post-surgery platelet count surge
Postoperative day 4	198,000	+30,000	Continued increase in platelet count post-surgery

However, the patient was readmitted four days after discharge for an urgent splenectomy due to persistent thrombocytopenia with a platelet drop to 14,000 × 10^9^/L. Preoperative preparations included 10 units of platelets, IV tranexamic acid, and IV fluids. The surgical procedure proceeded without complications. In the immediate postoperative period, her platelet count surged from 14,000 × 10^9^/L on the day of surgery to 168,000 × 10^9^/L by postoperative day 3, and further to 198,000 × 10^9^/L by postoperative day 4, without any signs of fever. The pathology report of the splenectomy corroborated the clinical diagnosis of ITP, noting red pulp expansion and hyperplasia.

Before discharge, the patient was educated and strongly advised on crucial preventive measures to mitigate her lifelong elevated risk of infections. She was counseled to receive essential vaccinations, including the 13-valent and 23-valent pneumococcal vaccines, quadrivalent and recombinant meningococcal B vaccines, the Hib vaccine, and the annual influenza vaccine to safeguard against overwhelming post-splenectomy infections. Always carrying a supply of high-dose antibiotics for self-administration in emergencies, recognizing signs of severe infections, and notifying healthcare providers about her splenectomy were emphasized. Prior to traveling, especially to malaria-endemic regions, seeking medical advice is imperative. Participation in a clinical registry for asplenic patients was strongly endorsed for continued education, management, and support. Adherence to these recommendations and consistent follow-up appointments are vital for her long-term health and well-being.

## Discussion

ITP manifests as an autoimmune disorder, with autoantibodies precipitating the early breakdown of platelets, leading to diminished platelet counts. The spleen and the mononuclear phagocyte system play pivotal roles in this process [10]. Individuals diagnosed with ITP exhibit a spectrum of symptoms, ranging from none at all to mild bruising and mucosal bleeding, and extending to severe bleeding episodes. However, symptomatic bleeding is rare, primarily occurring in cases where platelet counts fall below 30,000/mL, indicative of advanced stages of ITP [11,12].

In the case presented here, the patient diagnosed with ITP developed diffuse alveolar hemorrhage (DAH), a condition rarely associated with ITP. DAH results from damage to the alveolar microcirculation, often due to immune-related processes. It presents with symptoms such as dyspnea, hemoptysis, anemia, and radiological signs of diffuse alveolar filling. The DAH in our patient likely arose in connection with acute ITP, as suggested by the absence of other autoantibodies and the patient's immediate response to high-dose IVIG and corticosteroid therapy [4,13].

Various disorders, including different vasculitides and collagen vascular diseases, may also lead to DAH. Therefore, careful clinical evaluation is imperative when patients with ITP present with respiratory symptoms suggestive of DAH. DAH associated with ITP is treated with high-dose IVIG therapy, corticosteroids, and occasionally platelet transfusions. IVIG therapy has proven to be an effective first-line emergency treatment [1,14].

Additionally, the subject of this case displayed a form of ITP that was unresponsive to corticosteroids, necessitating alternative therapeutic interventions. Adults enduring corticosteroid-resistant or corticosteroid-dependent ITP for over three months might benefit from thrombopoietin receptor agonists (TPO-RAs), such as romiplostim or eltrombopag, depending on their preference for specific administration methods. Studies comparing these agents have found them to be similarly effective in sustaining response, preventing bleeding episodes, and reducing corticosteroid use with minimal variation [15-17].

For patients whose ITP does not respond to corticosteroids and persists for over three months, TPO-RA is often preferred over splenectomy. Splenectomy is typically delayed for at least a year due to the possibility of spontaneous remission. If ITP continues for more than 12 months, both splenectomy and TPO-RA are considered viable options, with the patient's preferences, age, overall health, and desire for either a durable response or long-term treatment influencing the decision-making process [18-28].

With splenectomy's decline as a favored second-line treatment due to postoperative complications, especially in older patients, alternatives such as TPO-RA are gaining prominence. TPO-RAs are reserved for patients unresponsive to other medical treatments, in accordance with international guidelines. The decision between TPO-RA and splenectomy should be individualized, taking into account various factors such as age, comorbidities, lifestyle, treatment compliance, and patient preferences [29,30].

In the presented case, the patient underwent elective splenectomy after initial treatments for refractory ITP proved partially successful. The procedure led to a significant improvement in platelet counts, aligning with pathology findings indicative of ITP. The clinical decisions made during the treatment course reflected the complex interplay of the patient's symptoms, ITP and DAH progression, and responses to different interventions, highlighting the necessity for individualized treatment approaches in managing ITP and its complications.

## Conclusions

ITP is a complex disorder exhibiting a range of clinical presentations. Its rare association with DAH poses significant challenges for clinicians, underscoring the need for increased awareness and careful clinical management. The case presented here underscores the importance of individualized treatment, demonstrating the effectiveness of various therapeutic approaches for refractory ITP. The choice between medical management and surgical interventions, such as splenectomy, should be based on a comprehensive clinical evaluation, taking into account the patient's overall presentation, disease progression, and response to treatment.
